# Metagenomic next-generation sequencing in the diagnosis of severe pneumonias caused by *Chlamydia psittaci*

**DOI:** 10.1007/s15010-020-01429-0

**Published:** 2020-04-20

**Authors:** Xiancheng Chen, Ke Cao, Yu Wei, Yajun Qian, Jing Liang, Danjiang Dong, Jian Tang, Zhanghua Zhu, Qin Gu, Wenkui Yu

**Affiliations:** 1 Department of Critical Care Medicine, Nanjing Drum Tower Hospital, The Affiliated Hospital of Nanjing University Medical School, No. 321 Zhongshan Road, Nanjing, 210008 Jiangsu Province China; 2 Department of Rheumatology and Immunology, Nanjing Drum Tower Hospital, The Affiliated Hospital of Nanjing University Medical School, Nanjing, China; 3 Department of Radiology, Nanjing Drum Tower Hospital, The Affiliated Hospital of Nanjing University Medical School, Nanjing, China

**Keywords:** Pneumonia, Psittacosis, *Chlamydia psittaci*, Untargeted next-generation sequencing, Tetracyclines

## Abstract

**Purpose:**

*Chlamydia psittaci* infection in humans can lead to serious clinical manifestations, including severe pneumonia, adult respiratory distress syndrome, and, rarely, death. Implementation of metagenomic next-generation sequencing (mNGS) gives a promising new tool for diagnosis. The clinical spectrum of severe psittacosis pneumonia is described to provide physicians with a better understanding and to highlight the rarity and severity of severe psittacosis pneumonia*.*

**Methods:**

Nine cases of severe psittacosis pneumonia were diagnosed using mNGS. Retrospective analysis of the data on disease progression, new diagnosis tool, treatments, and outcomes, and the findings were summarised.

**Results:**

Frequent symptoms included chills and remittent fever (100%), cough and hypodynamia (100%), and headache and myalgia (77.8%). All patients were severe psittacosis pneumonia developed respiratory failure, accompanied by sepsis in 6/9 patients. mNGS takes 48–72 h to provide the results, and help to identify diagnosis of psittacosis. Laboratory data showed normal or slightly increased leucocytes, neutrophils, and procalcitonin but high C-reactive protein levels. Computed tomography revealed air-space consolidation and ground-glass opacity, which began in the upper lobe of one lung, and spread to both lungs, along with miliary, nodular, or consolidated shadows. One patient died because of secondary infection with *Klebsiella pneumoniae*, while the other eight patients experienced complete recoveries.

**Conclusions:**

The use of mNGS can improve accuracy and reduce the delay in diagnosis of psittacosis. Severe psittacosis pneumonia responds well to the timely use of appropriate antibiotics.

## Introduction

*Chlamydia psittaci* infection in humans, known as parrot fever, ornithosis, or psittacosis, usually manifests as pneumonia, which ranges in severity from asymptomatic to fatal [1, 2]. *Chlamydia psittaci* is responsible for less than 5% of community-acquired pneumonia [3, 4]. Several outbreaks of *C. psittaci* infections in humans have occurred in different countries over the past 20 years, with a case fatality rate of less than 1% [5, 6]. *Chlamydia psittaci* enters the body mainly through the respiratory tract by inhaling aerosolised bacteria when exposed to infected secretions, droppings, or feathers [6]. *Chlamydia psittaci* occurs not only in Psittacinae and pigeons but also in poultry. Poultry, including chickens and ducks, are the most important sources of infection in China. One study found that the prevalence of *C. psittaci* in poultry sold in markets was 13% in chickens, 39% in ducks, and 31% in pigeons [7].

Contact with birds or poultry is regarded as the main risk factor for psittacosis, although it has been reported that 27% of patients do not have direct avian exposure [2]. The flu-like atypical pneumonia symptoms and an exposure history to birds are the primary criteria for clinical diagnosis. Laboratory diagnosis requires meeting any one of three criteria: (1) isolation of *Chlamydia psittaci* from respiratory secretions; (2) a fourfold or greater increase in antibody titre between serum samples collected 2 weeks apart, using a complement-fixation test (CFT) or micro-immunofluorescence (MIF); and (3) IgM antibody against *C. psittaci* titre detected by MIF of 1:16 or higher [5]. Polymerase chain reaction (PCR) is a faster and more specific diagnostic test, available in specialised diagnostic laboratories [8]. Because of its non-specific symptoms and the limitations of current tests, psittacosis is easily underdiagnosed and misdiagnosed [9].

Metagenomic next-generation sequencing (mNGS) is a new tool, which can rapidly and precisely identify potential pathogens, regardless of whether they are viral, bacterial, fungal, or parasitic [10]. Recent work has highlighted mNGS is the most promising approach for the comprehensive diagnosis of infections, particularly for severe pneumonia in intensive-care unit (ICU) settings [11].

Herein, we describe the clinical features of severe psittacosis pneumonia diagnosed by mNGS, and demonstrate that mNGS is an effective method for establishing the diagnosis. To our knowledge, there has been no similar study focusing on severe psittacosis pneumonia conducted in the recent years.

## Patients and methods

### Study design

We conducted a retrospective case review of nine patients admitted to Nanjing Drum Tower Hospital, a tertiary hospital in Nanjing, China, with severe psittacosis pneumonia between November 2017 and June 2019. For each case, data on prodromal symptoms, illness severity, dynamic and comprehensive computed tomography, and clinical course of the disease were extracted from electronic medical records. Additional data on the treatment, response to treatment, outcomes, and any relevant follow-up data were also collected.

The study protocol was approved by the Ethics Committee of the Nanjing Drum Tower Hospital, (Number 2019-183-01), and all data were anonymised prior to analysis. The study was conducted in compliance with the Declaration of Helsinki.

mNGS was conducted using the following operational steps [12, 13]

1. Clinical samples (blood or alveolar lavage fluid) were collected by following the standards of aseptic processing procedures. Nucleic acid extraction was conducted using TIANamp Micro DNA Kit (DP316, Tiangen Biotech, Beijing, China). 2. A total of 100 ng of the extracted DNA were subjected to processes of interruption, end repair, library construction, and sequencing. Agilent 2100 was used for quality control. Sequencing were performed at BGISEQ-100 platform (Beijing Genomics Institute, Wuhan, China). 3. The remaining nonhuman sequences were read after subtraction of the human host sequences mapped to the human reference genome (hg19) using Burrows–Wheeler Alignment and the low-quality reads and short reads (length < 35 bp) were removed. The remaining data were aligned to the four Microbial Genome Databases, consisting of bacteria, virus, fungi, and parasites. The mapped data were processed for advanced data analysis. Lists of suspected pathogenic microorganisms were produced, which included the numbers of strictly mapped reads, coverage rate, and depth. The clinical diagnosis was determined by considering all the clinical manifestations, possible pathogens identified by mNGS and other laboratory tests together.

### Diagnostic criteria for severe psittacosis pneumonia

To be included in the review, patients diagnosed with severe psittacosis pneumonia had to fulfil the following three criteria: (1) meet the criteria for severe community-acquired pneumonia [14]; (2) have specific fragment DNA of *C. psittaci* identified using mNGS; and (3) have negative results for all the routine etiological pathogen tests, including blood, sputum, and bronchoalveolar lavage fluid culture, and no other causative organism identified.

## Results

### Patient characteristics

Three women and six men with severe *C. psittaci* pneumonia were identified. Their median age was 64 (range 44–83) years (Table 1). All patients were positive for *C. psittaci* DNA fragments by mNGS, and systematic screening did not reveal any other respiratory pathogens on admission to our hospital. Regarding the exposure history, three patients had a definite exposure history, because they had all raised ducks or pigeon at home privately for years. Four patients had a contact history such as frequent visits to live poultry markets, and slaughtering live poultry for cooking. The remaining two patients had not had direct exposure to birds or poultry. It is noteworthy that seven of the nine patients (77.8%) patients had a history of exposure to, or close contact with, birds or poultry.

**Table 1:** Clinical characteristics of the severe psittacosis pneumonia cases

Characteristics	Patients, *n* (%)	Median value, (range)
Demographics		
Male/female	6/3	
Age, median (range, years)		64 (44–83)
History of contact with avian or poultry	7/9 (66.7)	
Underlying disease	8/9 (88.9)	
Clinical manifestations		
Fever > 38.5 °C	9/9 (100.0)	39.7 (39.0–40.5)
Cough, hypodynamia, dyspnoea	9/9 (100.0)	
Headache	7/9 (77.8)	
Myalgia	7/9 (77.8)	
Septic shock	6/9 (66.7)	
Invasive ventilator support	6/9 (66.7)	
APACHE II		23 (16–31)
Days from illness to respiratory failure		8 (2–10)
Laboratory testing		
Elevated WBC (normal 4–10, × 10^9^/L)	4/9 (44.4)	11.9 (5.5–22.0)
Elevated percentage of neutrophils (normal 45–75%)	7/9 (77.8)	82.4% (72.5–97.6%)
Elevated CRP (normal 0–8 mg/L)	9/9 (100.0)	175.0 (84.5–284.9)
Increased PCT (normal 0–0.5 ng/mL)	8/9 (88.9)	0.9 (0.2–2.7)
Elevated CK (normal 30–135 U/L)	4/8 (50.0)	831.0 (35.0–5179.0)
Elevated LDH (normal 109–245 U/L)	8/8 (100.0)	697.0 (357.0–1895.0)
Hypokalemia (normal 3.5–5.2 mmol/L)	6/9 (66.7)	3.4 (2.7–4.3)
Imaging		
Lesion began in superior lobe of lung	8/9 (88.9)	
Consolidation with air bronchograms	9/9 (100.0)	
Complete CT recovery in survivors	8/8 (100.0)	

*APACHE* The Acute Physiology and Chronic Health Evaluation, *CK* creatine kinase, *CRP* C-reactive protein, *CT* computed tomography, *LDH* lactate dehydrogenase, *PCT* procalcitonin, *WBC* white blood cell

All patients had chills, remittent fever higher than 39 °C, cough, weakness, and dyspnoea; seven patients had headache, cough producing white phlegm, and myalgia at the onset of their illness. These symptoms were followed by progressive dyspnoea. Five patients had lethargy or a more severe disturbance of consciousness, and two patients were in coma on admission to our unit. The median time from the onset of the illness to admission to our unit was 6 (range 2–11) days. The onset of respiratory failure was generally gradual, with a median time of 8 (range 2–10) days from the onset of the illness to respiratory failure. Six of the nine patients had septic shock and respiratory failure on admission, with mean Acute Physiology and Chronic Health Evaluation and Sequential Organ Failure Assessment scores of 23 and 6, respectively.

The clinical signs on physical examination of the patients were heterogeneous and non-specific, and included weakened respiratory sounds and wet rales on auscultation, with increased tactile vocal fremitus on the affected side. All patients developed respiratory failure and required respiratory support. Three patients improved after only using non-invasive ventilator, with a median use time of 4 (range 2–6) days. Six patients needed invasive ventilator ventilation, with a median ventilation time of 15 (range 7–17) days. The median ICU stay time was 25 (range 7–40) days.

### Technical investigations

On admission, the patients had a mean white blood cell count of 11.9 × 10^9^/L, percentage of neutrophils, 82.4%, C-reactive protein (CRP) level of 175 mg/L, and procalcitonin (PCT) level of 0.85 ng/mL. Six patients had elevated lactate dehydrogenase levels and four had elevated creatine kinase levels. In addition, five patients had hypokalaemia, and three had elevated aspartate aminotransferase and alanine aminotransferase levels.

**Fig. 1:**
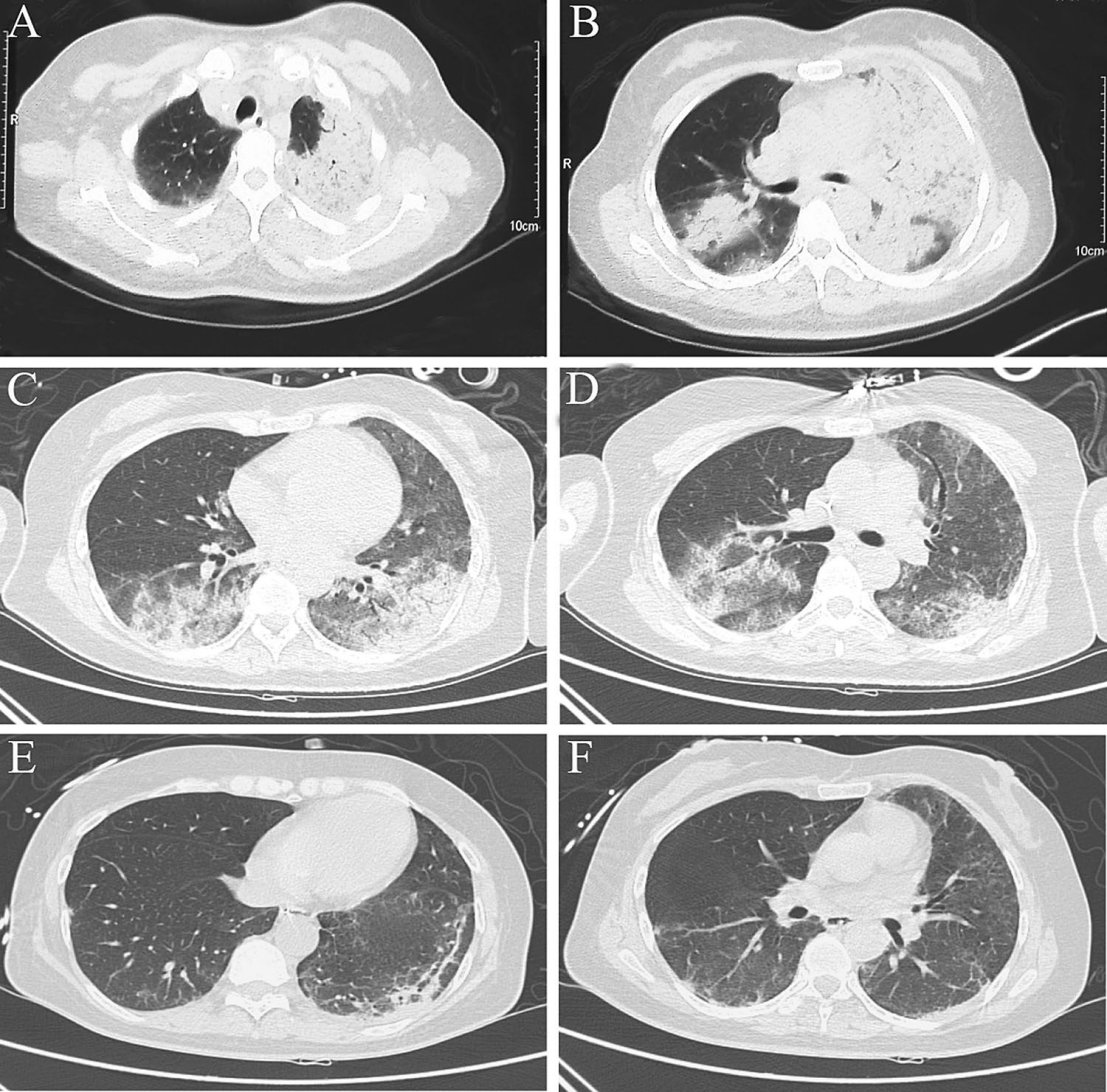
Serial chest computed tomography (CT) scans of a 49-year-old woman with severe psittacosis pneumonia. The initial CT scan (10 days after onset) shows air-space consolidation with inflammatory exudation in the superior lobe of the left lung and inferior lobe of the right lung (**a**, **b**). CT scan (19 days after the onset) shows that the consolidation area gradually decreased following treatment with extracorporeal membrane oxygenation (**c**, **d**). On follow-up, the consolidation disappeared, 26 days after the onset (**e**, **f**)

**Fig. 2:**
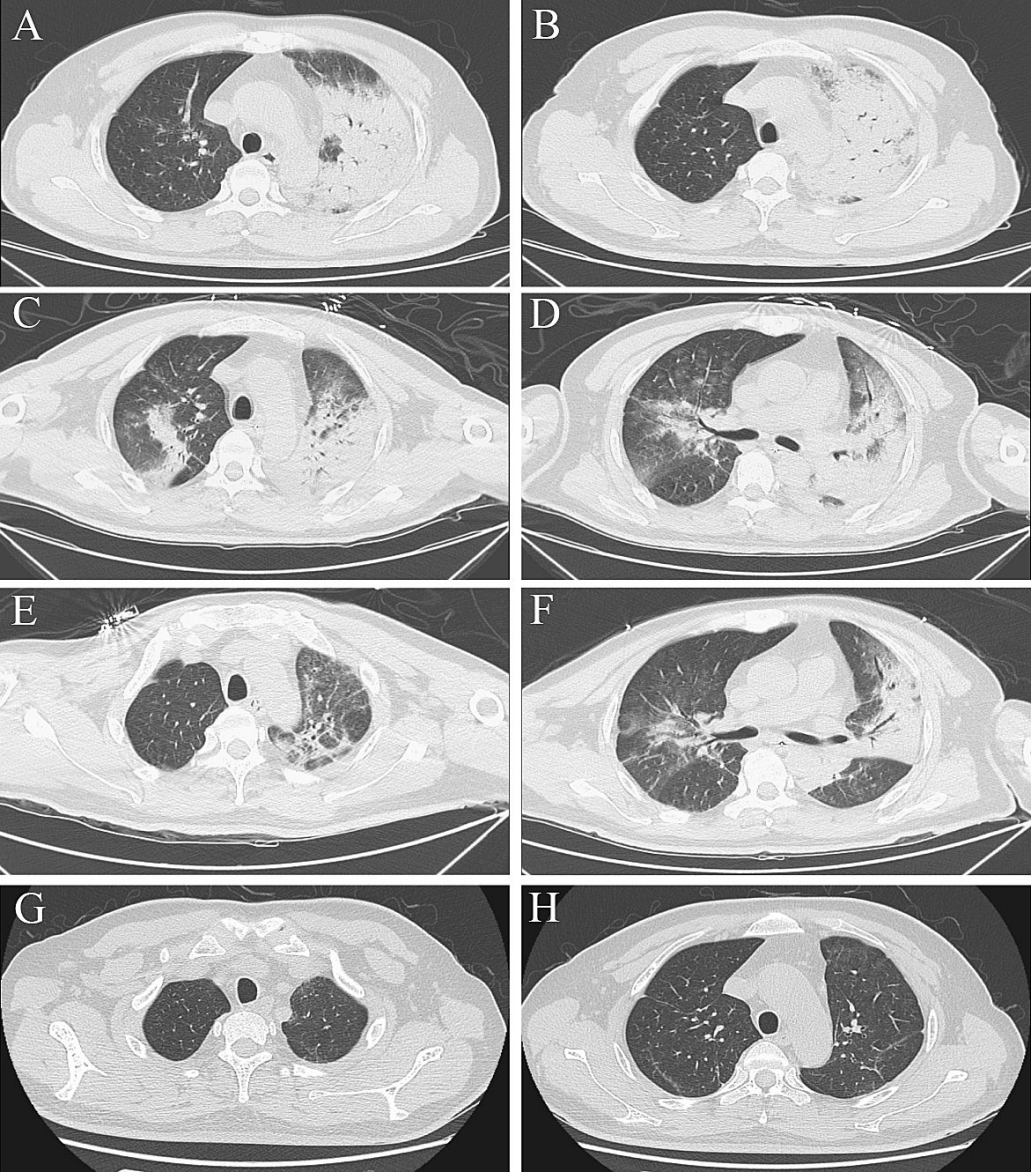
Serial chest computed tomography (CT) scans of a 43-year-old male farmer with severe psittacosis pneumonia. The initial CT scan (7 days after the onset) shows air-space consolidation with inflammatory exudation only appears in the superior lobe of left lung (**a**, **b**). The follow-up CT scan (16 days after the onset) shows exacerbation of the consolidated area in left lung and also in the middle and inferior lobes of right lung (**c**, **d**). On the follow-up CT scan (23 days after the onset), the area of consolidation has decreased (**e**, **f**) and 50 days after onset it has disappeared (**g**, **h**)

The inflammatory lesions were usually first observed in the upper lobe of lung. With the progression of psittacosis, the lobes were at times involved bilaterally. Air-space consolidation and ground-glass opacity, along with miliary, nodular, or consolidated shadows could be detected on computed tomography (CT) scan. Moreover, pleural effusions were also found in four of the nine patients (Figs. 1, 2). After clinical recovery, the inflammatory lesions gradually disappeared, with no residual fibrosis.

### Treatment

**Fig. 3:**
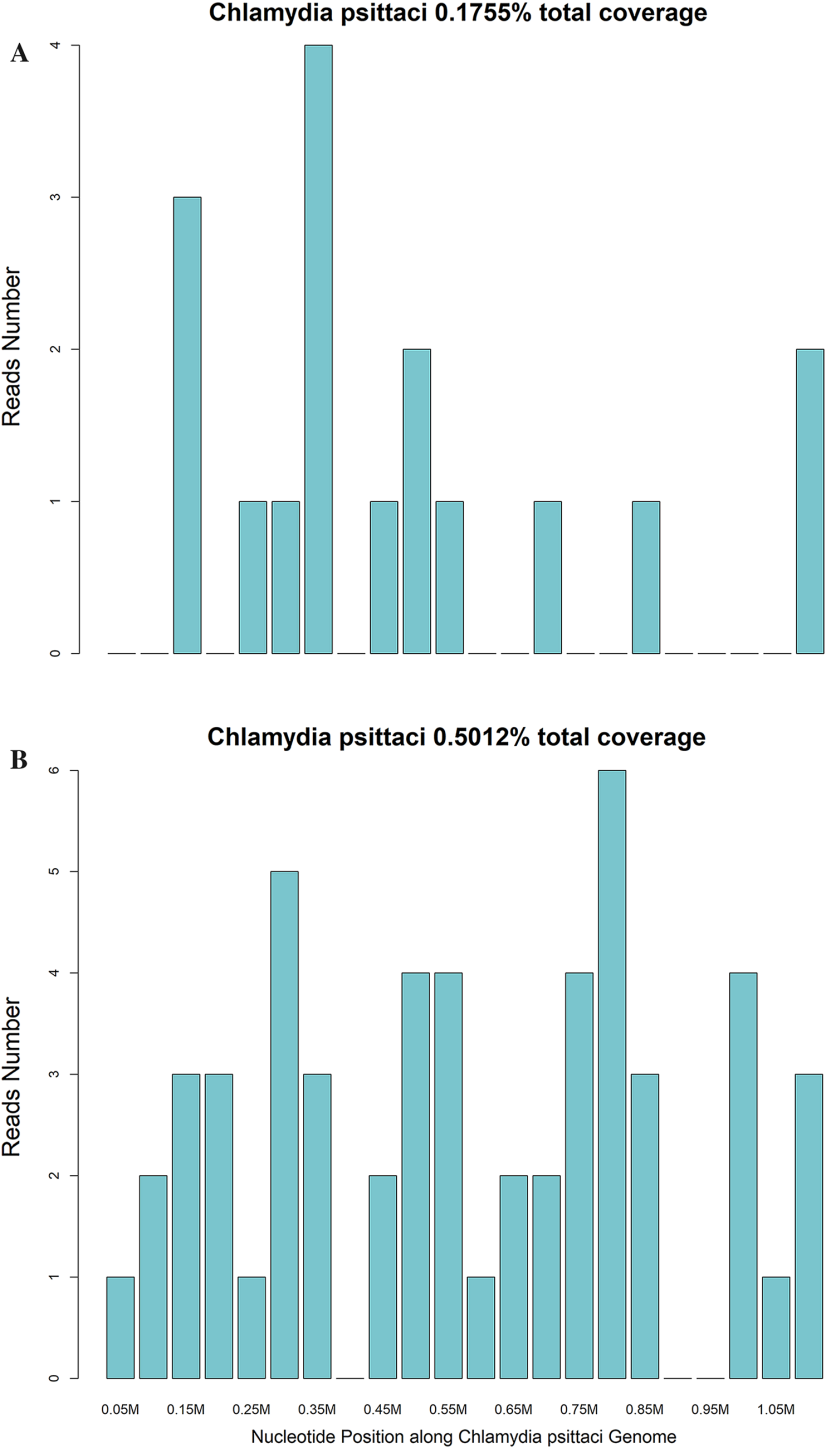
Metagenomic next-generation sequencing results of a 43-year-old male farmer with severe psittacosis pneumonia. **a** 17 specific *C. psittaci* gene fragments detected by mNGS in the blood sample, and non-repetitive fragments cover 0.1755% of the total *C. psittaci* gene, **b** 54 specific *C. psittaci* gene fragments detected by mNGS in in the alveolar lavage fluid, and non-repetitive fragments cover 0.5012% of the total *C. psittaci* gene

Prior to admission, patients came to community hospitals for help, the initial treatment was antipyretics such as paracetamol to control the symptoms, after symptoms exacerbated, clinicians in community hospitals added a cephalosporin (cefoxitin for three patients, cefdinir for two patients, and cefazolin for one patient) or ribavirin (one patient) depending on the result of the complete blood count; however, their clinical condition gradually deteriorated, leading to the development of acute respiratory distress syndrome. After admission to our hospital, patients underwent bronchofibroscopy, and alveolar lavage fluid was collected for mNGS. Six of the patients developed septic shock, and their blood and alveolar lavage fluid samples were sent for mNGS simultaneously (Fig. 3). The typical bronchofibroscopy findings were hyperaemic tracheal mucosa with oedema, a moderate amount of thin white or yellow secretions in the segmental bronchi, and occasional small patches of scattered haemorrhage.

Patients were started on empirical antibiotic therapy with β-lactam/β-lactamase inhibitor combinations and quinolones on ICU admission according to the community-acquired pneumonia management guideline [14]. The mNGS took 48–72 h from the receipt of the sample to the reporting of the results. When *C. psittaci* infection was confirmed, the antibiotic was changed to minocycline [2]. Patients with sepsis and protracted clinical courses were also treated with supplementary carbapenems, linezolid, or tigecycline. Minocycline was administered for at least 2 weeks, according to recommendations [2]**.**

### Outcomes

After minocycline therapy was initiated, patients’ fevers generally subsided within 3 days, and their respiratory function gradually improved. During the recuperation period, three patients had recurrent fever and increased inflammatory indicators, suggesting secondary infections in the lungs or bloodstream. One of these patients continued to have severe intestinal dysfunction, having developed *Klebsiella pneumoniae* bacteraemia, and eventually died of multidrug-resistant bacterial infection. The remaining eight patients experienced full recoveries.

## Discussion

We reported a retrospective study of the application of mNGS in the diagnosis of *C. psittaci* infection, manifesting as severe pneumonia, and the clinical features of *C. psittaci* infection. Our nine cases of severe psittacosis pneumonia, diagnosed with the aid of mNGS, and had characteristics and clinical features that differed from previously reported cases [7, 9]. Previous reports on psittacosis have included chills, white phlegm, cough, generalised myalgia, and weakness as general symptoms, and most reported cases have been of mild-to-middle severity [1, 2]. In our nine cases, headache was a prominent feature in seven patients, and five patients had lethargy or a coma, with one patient being suspected to have meningitis initially. Respiratory failure and septic shock developed gradually and necessitated the provision of mechanical ventilation and organ support therapy, which has not been reported previously. The patients’ laboratory data generally showed normal or slightly elevated leucocytes, neutrophils, and PCT, with high CRP levels. Knittler and Sachs [15] reported that *C. psittaci* is more pathogenic and multiplies more rapidly than other *Chlamydiales* species, and therefore, *C. psittaci* causes more severe inflammatory reactions. Inflammatory lesions in CT began in the upper lobe of lung, aggravated to bilateral lobes and air-space consolidation and ground-glass opacity, along with miliary, nodular, or consolidated shadows. Additionally, patients with septic shock and those who require mechanical ventilation are susceptible to secondary infections, which can lead to death, as illustrated by the patient in our study who died because of *Klebsiella pneumoniae* bacteraemia.

As *C. psittaci* belongs to the family *Chlamydiaceae* in the order *Chlamydiales* [16]*,* tetracyclines, macrolides, and quinolones, which can interfere with DNA and protein synthesis, can be used as antibiotic therapy [17]. Tetracyclines, including tetracycline hydrochloride and doxycycline, are generally regarded as the first-line treatment for psittacosis [18]. Minocycline, a second-generation tetracycline, also cures *C. psittaci* infections, with minimum inhibitory concentrations ranging from 0.03 to 0.06 mg/L and minimum bactericidal concentrations ranging from 0.06 to 0.25 mg/L in vitro [19]. Because of its higher efficacy and the lower incidence of side effects, our patients were treated with minocycline instead of tetracycline or doxycycline. Six of nine patients were started on a cephalosporin antibiotic initially; however, *C. psittaci* does not respond to cephalosporins, so this may have contributed to the severity of the pneumonia experienced by patients.

The methods for diagnosing *C. psittaci* infections have drawn attention for a long time, because until recently there has been no ideal tool. Isolation and culture of *C. psittaci* have a low efficiency and is hazardous for laboratory personnel [20]. Additionally, serology has cross-reactivity with other *Chlamydiaceae* species and is time-consuming [8]. PCR especially real-time PCR, give more rapid, sensitive, and specific way that can easily applied in laboratories where can undergo a real-time TaqMan-PCR [8, 21]. However, PCR for *C. psittaci* is unavailable in most hospitals in China, including many tertiary hospitals. PCR is a targeted test with a high specificity, but is only performed if clinicians suspect *C. psittaci* infection, which is difficult to diagnose clinically.

The advantage of mNGS is its wide detection range and the lack of need to specify the suspected causative organism a priori, and is used in the diagnosis of meningitis and encephalitis, and lower respiratory tract infections [11, 22]. In our hospital, the results of mNGS are available in 48–72 h, but routine sputum culture takes 5–7 days, and many cultures are negative. The ability of mNGS to obtain precise and timely microbial diagnoses of infections is a key advantage. When treating patients with severe pneumonia, physicians working in critical care need to identify the causal pathogen as early as possible, and to make an accurate diagnosis to provide targeted treatment. Because of the diagnostic challenges and its atypical clinical features, psittacosis is often misdiagnosed. It is worth using mNGS in patients with severe pneumonia to minimise the time to diagnosis of psittacosis and the course of the disease.

The main limitation of this study is that it included only nine cases of severe psittacosis pneumonia. This relatively small sample size is insufficient to investigate all the relevant features of psittacosis pneumonia. The study was a retrospective study and we did not use PCR, CFT, or MIF to confirm the diagnosis. In addition, this study was conducted in a central, provincial hospital; therefore, the findings may not be generalizable, because patients with milder illnesses would not have been admitted to our hospital.

## Conclusions

In our study, we diagnosed *C. psittaci* infection by mNGS and summarised the clinical features of severe psittacosis pneumonia. A history of exposure to poultry or birds and typical symptoms including chills, high fever, headache, and myalgia are important for diagnosis. Severe cases can slowly progress to respiratory failure and septic shock, and the death of one patient in this study indicates that *C. psittaci* can have serious consequences. Considering the poor response to empirical antibiotics and disease severity, mNGS can shorten the time to diagnosis and enable earlier initiation of targeted antibiotic therapy. Future studies should focus on simplifying the diagnosis, and the use serology application which is more widely available, as a screening test.
